# Effects of physical exercise on the lipid profile of perimenopausal and postmenopausal women: a systematic review and meta-analysis

**DOI:** 10.1590/1414-431X2025e14194

**Published:** 2025-03-03

**Authors:** J.V.M. Bernal, J.C. Sánchez-Delgado, A.M. Jácome-Hortúa, A.C. Veiga, G.V. Andrade, M.R. Rodrigues, H.C.D. de Souza

**Affiliations:** 1 Laboratório de Fisiologia e Fisioterapia Cardiovascular, Departamento de Ciências da Saúde, Faculdade de Medicina de Ribeirão Preto, Universidade de São Paulo, Ribeirão Preto, SP, Brasil; 2 Facultad de Ciencias Médicas y de la Salud, Universidad de Santander, Bucaramanga, Colombia; 3 Grupo de Investigación Ser Cultura y Movimiento, Facultad de Salud, Universidad Santo Tomás-Bucaramanga, Santander, Colombia

**Keywords:** Exercise, Secondary prevention, Review, Lipids, Climacteric, Menopause

## Abstract

During the climacteric period, the decline in ovarian hormones leads to changes in the lipid profile. Physical exercise is the main non-pharmacological recommendation for controlling lipid levels. However, the effects on the lipid profile in perimenopausal and postmenopausal women are incipient and inconclusive. In this context, we searched the Embase, PubMed, Scopus, and Web of Science databases for randomized clinical trials on the effects of exercise on the lipid profile of these women. We excluded studies that did not specify criteria for classifying the climacteric phase, that involved women undergoing hormone replacement therapy, or that examined combined treatments or acute effects of physical exercise. The meta-analysis indicated that general physical exercise increased high-density lipoprotein cholesterol (HDL-C) levels (mean difference [MD]=4.89; 95% confidence interval [95%CI]=0.97 to 8.81) in perimenopausal women. For obese postmenopausal women, 16 weeks of aerobic training increased HDL-C levels (MD=3.88; 95%CI=0.56 to 7.20) and reduced total cholesterol (MD=-22.36; 95%CI=-29.67 to -15.05) and low-density lipoprotein cholesterol (LDL-C) levels (MD=-17.86; 95%CI=-25.97 to -9.75), whereas 12 weeks of resistance training increased HDL-C levels (MD=4.20; 95%CI=1.16 to 7.23) and decreased triglycerides (MD=-14.86; 95%CI=-26.62 to -3.09) and LDL-C levels (MD=-16.36; 95%CI=-28.05 to -4.67). Overall, the results showed that physical exercise regulated lipid profiles in perimenopausal and postmenopausal women. Specifically, 12 weeks of resistance exercise and 16 weeks of aerobic exercise improved the lipid profile of obese postmenopausal women.

## Introduction

Cardiovascular diseases (CVDs) are the leading cause of death worldwide (1), with a high prevalence among middle-aged women (2). The cardiovascular changes observed during the climacteric period may explain this prevalence (2). The hormonal alterations during this period affect fat oxidation and distribution, lipid profile, and endothelial function and increase cardiac fibrosis and blood pressure in these women (3,4).

Regarding the lipid profile, although cardiometabolic alterations are already observed during perimenopause, studies often exclude women in this climacteric phase due to the high variability in ovarian follicular dysfunction (5). In contrast, postmenopausal women are frequently studied, and there is evidence that they have elevated levels of low-density lipoprotein cholesterol (LDL-C), total cholesterol, and triglycerides (6). Because an unfavorable lipid profile is associated with atherosclerosis and the development of CVDs, it is important to evaluate therapeutic interventions for this group of women to achieve greater benefits (5).

Among therapeutic options, the literature widely agrees that regular physical exercise provides numerous benefits for the cardiovascular system and has proven to be a crucial non-pharmacological therapeutic option (7,8). Specifically, evidence has shown that aerobic physical training (APT) generally reduces triglyceride levels (9) and increases high-density lipoprotein cholesterol (HDL-C) levels, protecting against atherosclerosis (10,11). In contrast, resistance exercise appears to significantly reduce the total cholesterol levels of postmenopausal women (12). Combined training programs that integrate aerobic and resistance exercises have been shown to be more effective in reducing insulin and LDL-C levels in overweight or obese individuals (13,14).

Indeed, different types of physical exercise can have different effects on cardiovascular health and lipid profiles. However, despite the relevance, there is a lack of systematic analyses or updated reviews describing the effects of different types of regular physical exercise on the lipid profile in perimenopausal and postmenopausal women. The present review aimed to fill this gap in the literature and contribute to a deeper understanding of the impact of various physical exercise modalities on the lipid profiles of perimenopausal and postmenopausal women.

## Material and Methods

This systematic review followed the Preferred Reporting Items for Systematic Reviews and Meta-analyses (PRISMA) guidelines (Supplementary Table S1) and was registered in PROSPERO (CRD42023417531).

### Search strategy

A systematic search for studies was conducted in the Embase, PubMed, Scopus, and Web of Science databases using a search strategy involving a combination of keywords extracted from Medical Subject Headings or EMTREE. The keywords used for the population were: climacteric, perimenopause, menopause, premature menopause, and postmenopause; for study type: randomized clinical trial, controlled clinical trial, and controlled clinical comparison; for intervention: exercise, physical exercise, exercise therapy, physical training, and resistance training; for outcomes: triglycerides, cholesterol, very low-density lipoproteins (VLDL-C), LDL-C, and HDL-C. These terms were combined using the Boolean operators “OR” and “AND”. The search strategy used in each database is described in Supplementary Table S2. In addition, we conducted a snowball search to track potential studies from the references and citations of previously included articles.

### Inclusion and exclusion criteria

The current review included randomized clinical trials published in English, Portuguese, or Spanish that investigated the effects of physical exercise on the lipid profile of perimenopausal or postmenopausal women. Perimenopause is characterized by the presence of irregular menstrual cycles during the period preceding the last menstruation, and postmenopause is defined by the absence of menstruation for more than 12 months (15). In contrast, studies that did not clearly specify the criteria used to classify the climacteric phase were excluded, as well as those involving animals, women undergoing chemotherapy, radiotherapy, or hormone replacement therapy, and those that addressed combined treatments or investigated the acute effects of physical exercise.

### Study selection and methodological quality

After the search, one of the authors (JVMBS) removed duplicate records. Subsequently, the title and abstract of the studies were assessed according to inclusion and exclusion criteria. The selected studies then underwent a full-text evaluation. The study selection process was conducted by two independent reviewers (JVMBS, AMJ-H), with a third reviewer (JCS-D) involved in cases of disagreement. The level of agreement between the two reviewers was determined using Cohen's Kappa value, with Jamovi software (version 2.3) (16,17).

The methodological quality of the included articles was assessed using the PEDro scale (available at www.pedro.org.au, accessed on August 10, 2024) (18-20). The PEDro scale scores range from 0 to 10 points, with higher scores indicating better methodological quality. Articles scoring between 9 and 10 points were classified as excellent, 6 to 8 points as good, 4 to 5 points as fair, and less than 4 points as poor methodological quality (21). Additionally, the GRADEPro online tool was used to determine the certainty or quality of evidence regarding the risk of bias, imprecision, inconsistency, indirectness, and publication bias.

### Data extraction and analysis

Finally, we extracted and analyzed information regarding sample size, age, type of intervention, and lipid profile outcomes found in the selected articles. To standardize the presentation of results, data presented in mmol/L were converted to mg/dL using the conversions: 1 mg/dL=0.0259 mmol/L for cholesterol and 1 mg/dL=0.0113 mmol/L for triglycerides (22). The original data in mmol/L and the standardized data in mg/dL are presented in Supplementary Tables S3 and S4, respectively. Data presented in graphical form were extracted using WebPlotDigitizer software, version 4.6 (USA). The synthesis and analysis of information were narrative and qualitative. When possible, meta-analyses were performed using RevMan 5.4 to compare mean differences (MD) and 95% confidence intervals (95%CI) for continuous variables between intervention and control/comparison groups.

## Results

### Included studies and population

**Figure 1:**
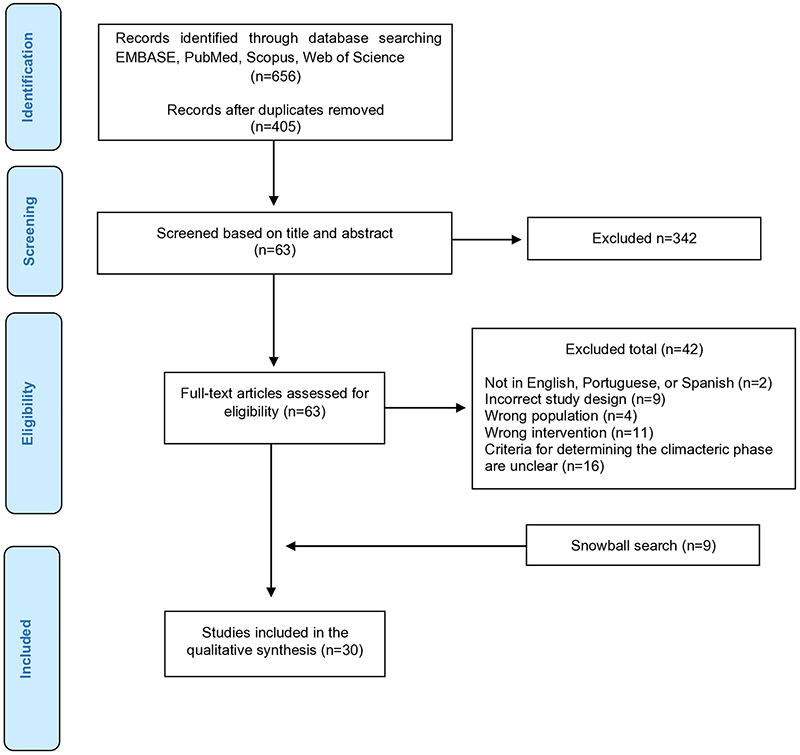
Flowchart of included studies.

The initial search found 656 trials. Of these, only 21 met the eligibility criteria and were included (23-43). Additionally, nine studies were added through the snowball search (44-52) (Figure 1). The agreement in study selection between the reviewers had a Kappa value of 0.79 for title and abstract screening and 0.83 for full-text evaluation. Of the included studies, two involved only perimenopausal women (23,24), 27 involved only postmenopausal women (25-42,44-52), and one involved both perimenopausal and postmenopausal women (43). The analyzed trials included 1,474 postmenopausal participants (880 in the exercise/intervention group and 594 in the control group) and 98 perimenopausal participants (64 in the exercise/intervention group and 34 in the control group).

### Methodological quality of studies and evidence quality

All studies included in the analysis adhered to the PEDro scale criteria, which required randomization, blinded distribution of subjects, initial and intergroup comparisons, and measures of variability for at least one key outcome. None of the studies implemented patient or assessor blinding. The identified articles scored between 4 and 8 on the PEDro scale (Supplementary Table S5). Regarding the quality of evidence from the meta-analysis results, we observed low certainty levels for the effect of physical exercise on HDL-C levels in perimenopausal women. In obese postmenopausal women, we found low certainty levels for the effects of 16 weeks of APT on triglyceride levels and moderate certainty levels for its effects on LDL-C and HDL-C levels. In contrast, we observed moderate certainty levels for the effects of 12 weeks of resistance training on triglyceride levels and low certainty levels for its effects on LDL-C and HDL-C levels in obese postmenopausal women (Supplementary Table S6).

### Study characteristics

The analyzed studies investigated the effects of different modalities of physical training. These included APT (n=15), resistance training (n=12), concurrent or combined training (n=5), and functional or sport-based training (n=4). Table 1 presents the characteristics of studies that included perimenopausal women, while Supplementary Table S7 presents the characteristics of studies that included postmenopausal women.

**Table 1:** Effect of physical exercise on the lipid profile of perimenopausal women.

Study (Country)	Participants’ characteristics	Participants at baseline (n)		Age (years)	Intervention (intensity)	Evaluated outcomes	Significant differences between groups
Krishnan et al. 2014 (23) (USA)	Healthy	CON	n=14	46.7±3.3	Maintained the level of physical activity as usual.	TG; Chol; LDL-C; HDL-C	No significant differences were observed. The analysis included Group CON (n=10) and Group EX (n=18).
		EX	n=21		Aerobics plus strength training (AT - 50 to 80% of HR reserve). D: 24; Ds: 60; F: 6.		
Blumenthal et al. 1991 (43) (USA)	Healthy	AT	n=12	42±3	Aerobic training (70% of the HR reserve). D: 12; Ds: 50; F: 3.	TG; Chol; LDL-C; HDL-C; VLDL-C; APO	No significant differences were observed. The analysis included Group AT (n=12) and Group ST (n=11).
		ST	n=11		Strength training (established by the maximum repetition zone - 12 to 15 repetitions). D: 12; Ds: 55; F: 2.		
Costa et al. 2018 (24) (Brazil)	Dyslipidemia	CON	n=20	46.8	Maintained normal habits.	TG; Chol; LDL-C; HDL-C; Chol/HDL	After training, the EX group showed a significant increase in HDL-C values and a significant reduction in Chol, LDL-C, and Chol/HDL values. Furthermore, after training, significant differences were observed between the groups regarding the values of Chol, LDL-C, and Chol/HDL; in all of them, the EX group presented lower values. The analysis included Group CON (n=14) and Group EX (n=16).
		EX	n=20	46.2	Water-based aerobic training (Borg scale - 9 to 15). D: 12; Ds: 45; F: 2.		

n: sample size at baseline; CON: control; EX: exercise; AT: aerobic training; ST: strength training; USA: United States of America; HR: heart rate; D: duration of the intervention (weeks); Ds: duration of the exercise session (minutes); F: exercise frequency (times/week); TG: triglycerides; Chol: total cholesterol; LDL-C: low-density lipoprotein cholesterol; HDL-C: high-density lipoprotein cholesterol; VLDL-C: very-low-density lipoprotein; APO: Apolipoprotein; Chol/HDL: ratio between Chol/HDL.

### Effects of physical exercise on the lipid profile of perimenopausal women

Three trials investigated physical exercise effects on the lipid profile of perimenopausal women (23,24,43). Among these, Costa et al. (24) reported that 12 weeks of water-based APT twice a week with intensity based on subjective effort perception increased HDL-C levels [mean (95%CI), baseline 47.69 (43.20; 52.18) *vs* post-intervention 52.44 (46.99; 57.88) mg/dL] and reduced total cholesterol levels [mean (95%CI), baseline 220.50 (206.94; 234.06) *vs* post-intervention 199.75 (187.51; 211.99) mg/dL], LDL levels [mean (95%CI), baseline 140.39 (127.58; 153.19) *vs* post-intervention 117.34 (104.59; 130.08) mg/dL], as well as the total cholesterol/HDL-C ratio [mean (95%CI), baseline 4.71 (4.41; 5.02) *vs* post-intervention 3.91 (3.62; 4.21)] in dyslipidemic women (Table 1).

**Figure 2:**
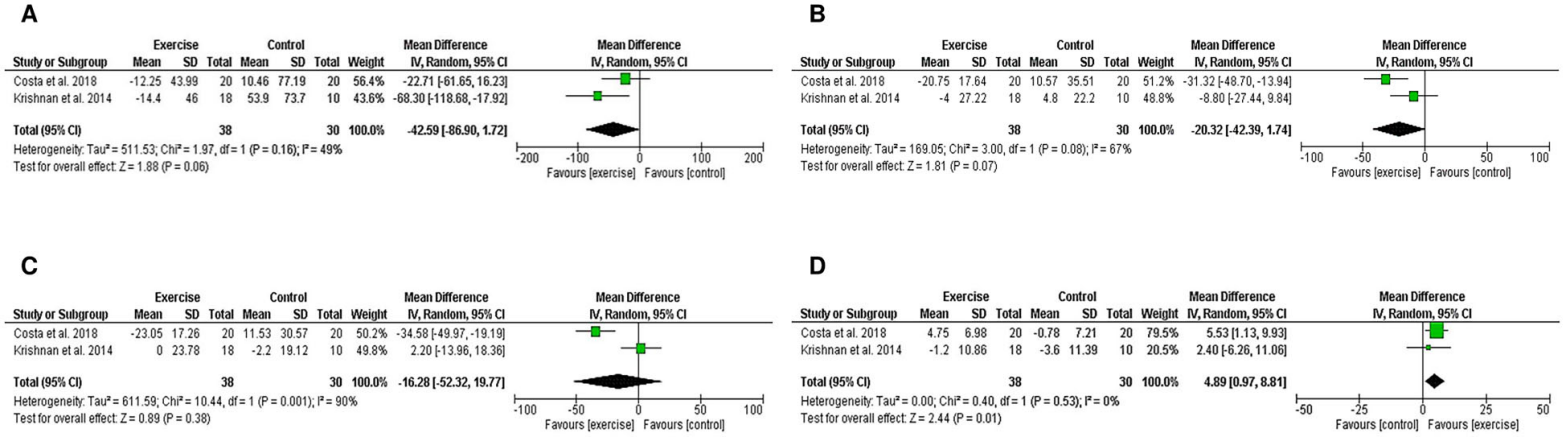
Forest plot of meta-analysis results presented as pooled mean differences with 95%CI for changes in triglycerides (**A**), total cholesterol (**B**), LDL-C (**C**), and HDL-C (**D**) for exercise and control groups. The effects of physical exercise on perimenopausal women are graphically represented with black diamonds. LDL-C: low-density lipoprotein cholesterol; HDL-C: high-density lipoprotein cholesterol. See references 23 and 24.

The meta-analysis indicated that physical exercise increased HDL-C levels (MD=4.89; 95%CI=0.97 to 8.81, P=0.01; I^2^=0%) in perimenopausal women (Figure 2).

### Effects of physical exercise on the lipid profile in postmenopausal women

#### APT

Thirteen trials investigated the effects of APT on lipid profile parameters in postmenopausal women (25,27,29,30,31,35,41-43,46,48,49,51). Of these, three investigated the effects of eight weeks of APT (27,46,48), two evaluated the impact of 12 weeks (41,43), five analyzed the effects of 16 weeks (25,29,30,49,51), and three examined the role of 24 weeks (31,35,42).

Regarding the effects of eight weeks of APT, Miyaki et al. (27) showed that eight weeks of APT three to five times per week and intensity at 60 to 75% of maximum heart rate increased HDL-C levels [means±SD, baseline 64.86±10.81 *vs* post-intervention 71.81±11.97 mg/dL]. Diniz et al. (46) demonstrated that eight weeks of APT with intensity at 100% of the critical velocity protocol reduced LDL-C levels [means±SD, Control Group (CON) 124.56±30.75 *vs* Experimental Group (EXP) 106.70±31.24 mg/dL]. Kazemi et al. (48) reported that eight weeks of APT three times per week and intensity of 80 to 90% of maximum heart rate increased HDL-C levels [means±SD, baseline 51.9±4.60 *vs* post-intervention 64.7±5.22 mg/dL] and reduced LDL-C levels [means±SD, baseline 116.8±19.79 *vs* post-intervention 85.7±15.47 mg/dL], triglycerides [means±SD, baseline 193.8±15.73 *vs* post-intervention 169.6±19.58 mg/dL], and total cholesterol [means±SD, baseline 207.4±12.78 *vs* post-intervention 180.6±17.36 mg/dL].

**Figure 3:**
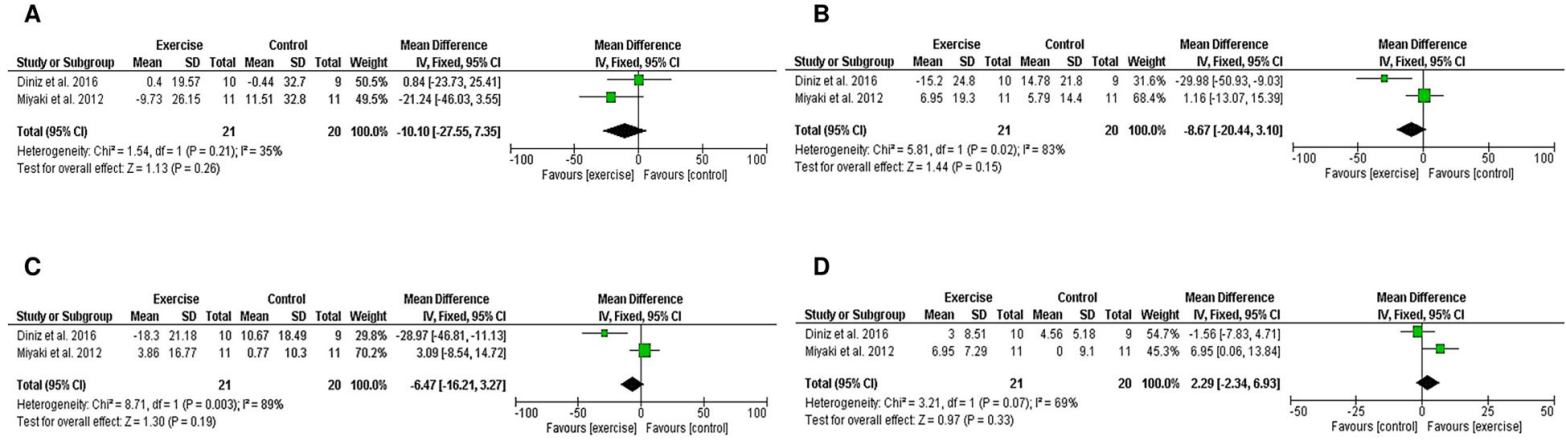
Forest plot of meta-analysis results presented as pooled mean differences with 95%CI for changes in triglycerides (**A**), total cholesterol (**B**), LDL-C (**C**), and HDL-C (**D**) for exercise and control groups. The effects of eight weeks of aerobic physical training on postmenopausal women are graphically represented with black diamonds. LDL-C: low-density lipoprotein cholesterol; HDL-C: high-density lipoprotein cholesterol. See references 27 and 46.

The meta-analysis revealed that eight weeks of APT did not significantly alter triglyceride levels (MD=-10.10; 95%CI=-27.55 to 7.35, P=0.26; I^2^=35%), total cholesterol (MD=-8.67; 95%CI=-20.44 to 3.10, P=0.15; I^2^=83%), LDL-C (MD=-6.47; 95%CI=-16.21 to 3.27, P=0.19; I^2^=89%), and HDL-C (MD=2.29; 95%CI=-2.34 to 6.93, P=0.33; I^2^=69%) in postmenopausal women (Figure 3).

Concerning the impact of 12 weeks of APT, Wang et al. (41) reported that 12 weeks of APT three times per week and intensity at 60 to 80% of heart rate reserve increased HDL-C levels [means±SD, baseline 46.2±11.3 *vs* post-intervention 50.7±11.6 mg/dL]. However, Blumenthal et al. (43) found that 12 weeks of APT with three times per week and intensity at 70% of heart rate reserve reduced HDL-C levels [means±SD, baseline 61±16 *vs* post-intervention 57±13 mg/dL].

In relation to the effects of 16 weeks of APT, Rossi et al. (25) and Rossi et al. (30) did not observe significant differences in lipid profiles after APT with an intensity of 100% of the critical velocity protocol. Moreover, Reis et al. (51) did not observe significant changes in lipid profiles after 16 weeks of aquatic exercise. Kim et al. (49) showed that 16 weeks of APT three times per week and intensity of 55 to 80% of maximum heart rate reduced total cholesterol levels [means±SD, CON 213.77±12.90 *vs* EXP 197.85±16.30 mg/dL]. Rossi et al. (29) reported that 16 weeks of APT with intensity at 100% of critical velocity protocol reduced the total cholesterol/HDL-C ratio [means±SD, baseline 3.6±0.9 *vs* post-intervention 3.4±0.8 mg/dL].

**Figure 4:**
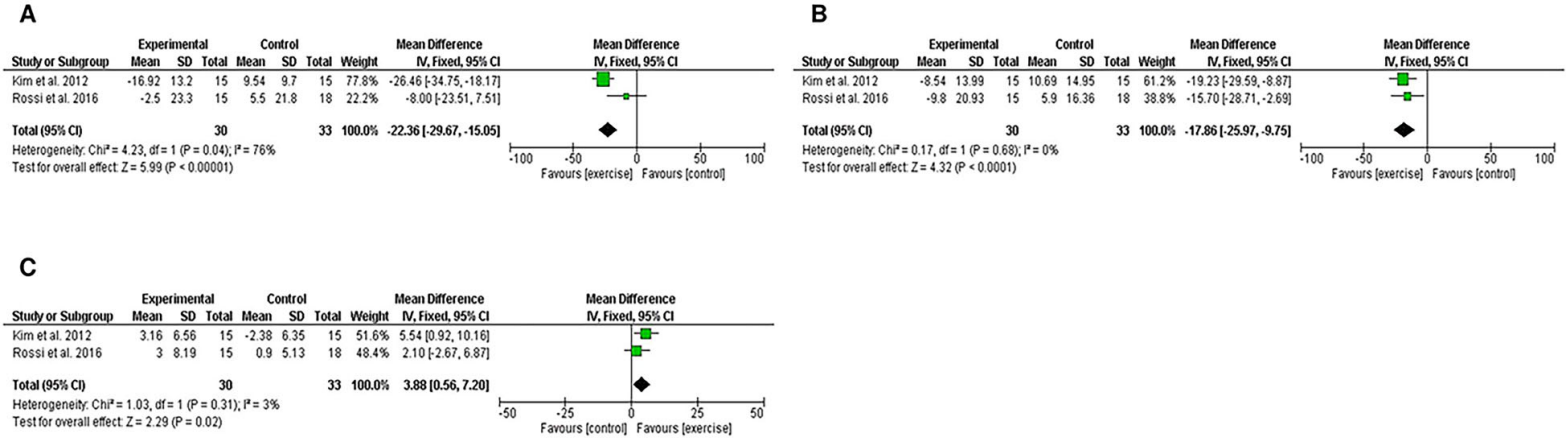
Forest plot of meta-analysis results presented as pooled mean differences with 95%CI for changes in total cholesterol (**A**), LDL-C (**B**), and HDL-C (**C**) for exercise and control groups. The effects of 16 weeks of aerobic physical training on obese postmenopausal women are graphically represented with black diamonds. LDL-C: low-density lipoprotein cholesterol; HDL-C: high-density lipoprotein cholesterol. See references 29 and 49.

The meta-analysis revealed that 16 weeks of APT increased HDL-C levels (MD=3.88; 95%CI=0.56 to 7.20, P=0.02; I^2^=3%) and reduced total cholesterol (MD=-22.36; 95%CI=-29.67 to -15.05, P<0.0001; I^2^=76%) and LDL-C levels (MD=-17.86; 95%CI=-25.97 to -9.75, P<0.0001; I^2^=0%) in obese postmenopausal women (Figure 4).

Regarding the effects of 24 weeks of APT, Cauley et al. (42) found no statistically significant differences in lipid profile after a walking program. In turn, Dash et al. (35) demonstrated that 24 weeks of APT three times per week and intensity at 45 to 65% of maximum VO_2_ increased HDL-C levels [mean change ±SE, Control Group (CON) -1.06±1.38 *vs* Experimental Group (EXP) +5.80±2.33 mg/dL] in women with a family history of breast cancer. Additionally, Dash et al. (35) also showed that 24 weeks of walking with moderate intensity reduced triglyceride levels [means±SE, CON +22.09±19.02 *vs* EXP -17.59±15.44 mg/dL] and increased HDL-C levels [mean change ±SE, CON -1.06±1.38 *vs* EXP +6.21±3.00 mg/dL] in women with a family history of breast cancer. In contrast, Ready et al. (31) showed that 24 weeks of walking with an intensity of 60% of the heart rate reserve reduced total cholesterol [means±SD, baseline 256.37±18.92 *vs* post-intervention 244.79±21.62 mg/dL], triglycerides [means±SD, baseline 159.29±87.61 *vs* post-intervention 148.67±82.30 mg/dL], and the total cholesterol/HDL-C ratio [means±SD, baseline 5.19±1.27 *vs* post-intervention 5.06±1.59] (Supplementary Table S7).

**Figure 5:**
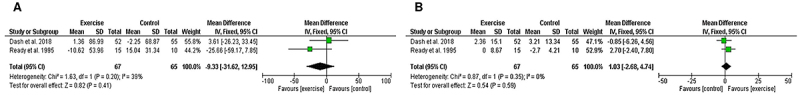
Forest plot of meta-analysis results presented as pooled mean differences with 95%CI for changes in triglycerides (**A**) and HDL-C (**B**) for exercise and control groups. The effects of 24 weeks of walking in postmenopausal women with cardiometabolic alterations are graphically represented with black diamonds. HDL-C: high-density lipoprotein cholesterol. See references 31 and 35.

The meta-analysis revealed that 24 weeks of walking did not alter triglyceride levels (MD=-9.33; 95%CI=-31.62 to 12.95, P=0.41; I^2^=39%) and HDL-C levels (MD=1.03; 95%CI=-2.68 to 4.74, P=0.59; I^2^=0%) in postmenopausal women with metabolic disorders (Figure 5).

#### Resistance training

Twelve trials investigated the effects of resistance training on lipid profile in postmenopausal women (28,33,36,37,39,40,43-45,47,48,52). Of these, two examined the effects of eight weeks resistance training (47,48), four evaluated the impact of 12 weeks (33,39,43,44), one analyzed the effects of 15 weeks (52), two investigated the effects of 16 weeks (36,37), two examined the effects of 24 weeks (40,45), and one evaluated the impact of 48 weeks (28).

Regarding the effects of eight weeks of resistance training, Elliott et al. (47) reported that eight weeks of resistance training three times per week and with progressive intensity did not significantly alter the lipid profile. In turn, Kazemi et al. (48) demonstrated that eight weeks of resistance training three times per week and with intensity of 75% of one repetition maximum increased HDL-C levels [means±SD, baseline 50.2±3.39 *vs* post-intervention 62.7±4.47 mg/dL] and reduced LDL-C levels [means±SD, baseline 105.5±23.38 *vs* post-intervention 86.8±13.81 mg/dL], triglycerides [means±SD, baseline 192.4±19.29 *vs* post-intervention 171.9±16.33 mg/dL], and total cholesterol [means±SD, baseline 205.8±9.06 *vs* post-intervention 188.8±9.06 mg/dL].

Concerning the impact of 12 weeks of resistance training, Blumenthal et al. (43) and Cardoso et al. (44) observed no significant differences in the lipid profile after eight weeks of resistance training. Blumenthal et al. (43) used a frequency of twice per week with intensity established by the repetition maximum zone, while Cardoso et al. (44) applied a frequency of three to five times per week with intensity ranging from 50 to 80% of one repetition maximum. In turn, Wooten et al. (39) reported that 12 weeks of resistance training at a frequency of three times per week and progressive intensity reduced levels of total cholesterol [means±SE, baseline (+24 h) 205.02±15.44 *vs* post-intervention (+24 h) 164.48±15.83 mg/dL] and LDL-C [means±SE, baseline (+24 h) 130.89±12.74 *vs* post-intervention (+24 h) 94.59±11.58 mg/dL]. Son et al. (33) showed that 12 weeks of resistance training at a frequency of three times per week and intensity of 40 to 70% of one-repetition maximum increased HDL-C levels [mean change±SD, 5.1±0.9 mg/dL) and reduced triglyceride levels [mean change±SD, 9.4±3.0 mg/dL) and LDL-C [mean change±SD, -10.8±5.3 mg/dL].

**Figure 6:**
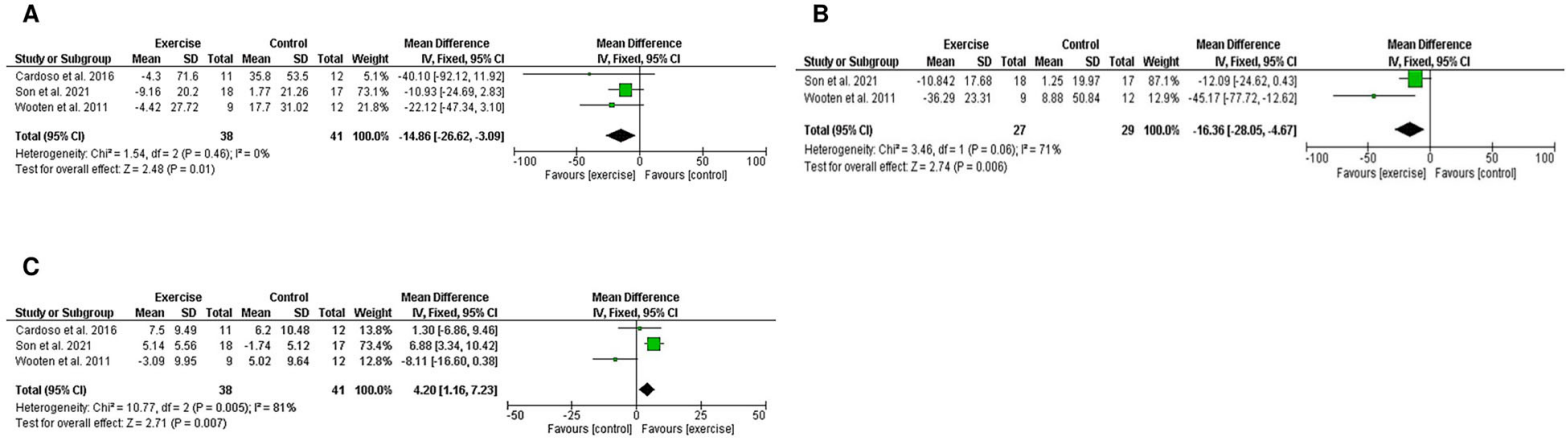
Forest plot of meta-analysis results presented as pooled mean differences with 95%CI for changes in triglycerides (**A**), LDL-C (**B**), and HDL-C (**C**) for exercise and control groups. The effects of 12 weeks of resistance exercise in obese postmenopausal women are graphically represented with black diamonds. LDL-C: low-density lipoprotein cholesterol; HDL-C: high-density lipoprotein cholesterol. See references 33, 39, and 44.

The meta-analysis revealed that 12 weeks of resistance exercise increased HDL-C levels (MD=4.20; 95%CI=1.16 to 7.23, P=0.007; I^2^=81%) and reduced triglyceride levels (MD=-14.86; 95%CI=-26.62 to -3.09, P=0.01; I^2^=0%) and LDL-C levels (MD=-16.36; 95%CI=-28.05 to -4.67, P=0.006; I^2^=71%) in obese postmenopausal women (Figure 6).

In relation to the effects of 15 and 16 weeks of resistance training, Ward et al. (52) reported that 15 weeks of resistance training at a frequency of three times per week and progressive intensity reduced total cholesterol levels [median (interquartile range), baseline 216.22 (185.33; 254.83) *vs* post-intervention 204.63 (169.88; 250.96) mg/dL] and LDL-C levels [median (interquartile range), baseline 111.96 (100.39; 158.30) *vs* post-intervention 111.96 (92.66; 158.30) mg/dL]. Conceição et al. (36) observed no significant differences in the lipid profile after 16 weeks of resistance training at a frequency of three times per week and intensity determined by the repetition maximum zone. In turn, Libardi et al. (37) demonstrated that 16 weeks of resistance training at a frequency of three times per week and intensity established by the maximal repetition zone reduced levels of total cholesterol [means±SD, baseline 223.95±69.60 *vs* post-intervention 183.21±27.06 mg/dL] and LDL-C [means±SD, baseline 145.13±41.28 *vs* post-intervention 85.86±27.59 mg/dL].

Regarding the effects of 24 and 48 weeks of resistance training, Colado et al. (45) reported that 24 weeks of resistance training using elastic bands at a frequency of two to three times per week increased HDL-C levels [means±SD, baseline 64.1±11.9 *vs* post-intervention 72±9.7 mg/dL] and reduced the total cholesterol/HDL-C ratio [means±SD, baseline 3.6±0.7 *vs* post-intervention 3.2±0.7]. In turn, Rodrigo et al. (40) demonstrated that 24 weeks of resistance training at a frequency of two to three times per week and progressive intensity increased HDL-C values [means±SD, baseline 66.40±11.49 *vs* post-intervention 69.86±10.94 mg/dL]. However, it is worth noting that the control group in the study mentioned above, which received general recommendations on exercise and nutrition, showed higher HDL-C values [means±SD, baseline 64.27±11.67 *vs* post-intervention 69.24±8.41 mg/dL] after the intervention period (25) (Supplementary Table S7). Additionally, Gómez-Tomás et al. (28) showed that 48 weeks of resistance training at a frequency of three times per week and progressive intensity reduced levels of total cholesterol [means±SD, baseline 222.72±42.88 *vs* post-intervention 207.00±34.77 mg/dL] and LDL-C [means±SD, baseline 142.54±35.86 *vs* post-intervention 125.78±33.15 mg/dL] (Supplementary Table S7).

#### Concurrent or combined training

Four trials investigated the effects of concurrent (25,30) or combined (29,50) training on lipid profile parameters in postmenopausal women. Of these, three demonstrated changes after the intervention (29,30,50). Machado et al. (50) reported that eight weeks of combined training at a frequency of three times per week and an intensity of 50 to 70% of maximum heart rate reduced LDL-C levels [means±SE, baseline 98±8.4 *vs* post-intervention 86.4±8.1 mg/dL]. The study also demonstrated that eight weeks of combined training at a frequency of three times per week and an intensity exceeding 70% of maximum heart rate increased HDL-C levels [means±SE, baseline 47.3±5.3 *vs* post-intervention 50.2±5.2 mg/dL]. In contrast, Rossi et al. (29) showed that 16 weeks of combined training at an intensity established by the maximal repetition zone increased HDL-C levels [means±SD, baseline 51.9±10.7 *vs* post-intervention 54.8±12.0 mg/dL] in overweight or obese women. In another study, Rossi et al. (30) demonstrated that the same training protocol described above increased HDL-C levels [means±SD, baseline 57.1±17.3 *vs* post-intervention 64.3±16.1 mg/dL; means±SD, baseline 44.7±9.6 *vs* post-intervention 50.3±15.3 mg/dL] and reduced the total cholesterol/HDL-C ratio [means±SD, baseline 3.6±0.9 *vs* post-intervention 3.0±0.6 mg/dL; means±SD, baseline 5.2±1.1 *vs* post-intervention 4.7±1.2 mg/dL] in women with normal triglyceride levels (triglycerides <150) and women with elevated triglyceride levels (triglycerides ≥150), respectively (Supplementary Table S7).

#### Functional or sport-based training

Four trials investigated the effects of functional or sport-based training (taekwondo, handball, or yoga) on lipid profile parameters in postmenopausal women (26,32,34,38). All of them demonstrated changes after the intervention. Neves et al. (26) showed that 16 weeks of functional training at a frequency of three times per week and intensity established by the critical velocity protocol reduced HDL-C levels [means±SD, baseline 58.44±15.5 *vs* post-intervention 52.56±15.3 mg/dL]. Lee et al. (32) demonstrated that 16 weeks of taekwondo at a frequency of five times per week reduced total cholesterol [means±SD, baseline 183.6±42.8 *vs* post-intervention 169.8±42.2 mg/dL] and LDL-C [means±SD, baseline 100.8±38.8 *vs* post-intervention 93.5±35.7 mg/dL]. Pereira et al. (34) showed that 16 weeks of handball-based exercises at a frequency of two to three times per week and intensity around 76±6% of maximum heart rate reduced total cholesterol levels [means±SD, baseline 216.22±30.89 *vs* post-intervention 212.35±30.89 mg/dL] and LDL-C [means±SD, baseline 139.0±34.75 *vs* post-intervention 131.27±30.89 mg/dL]. Finally, Lee et al. (38) showed that 16 weeks of yoga exercises at a frequency of three times per week reduced levels of total cholesterol [means±SD, CON 215.25±13.69 *vs* EXP 195.13±16.99 mg/dL] (Supplementary Table S7).

## Discussion

The narrative analysis indicated that APT can enhance the lipid profile of perimenopausal women with dyslipidemia. Additionally, various exercise modalities - including aerobic, resistance, combined, and functional/sports training - were found to have beneficial effects on the lipid profile of postmenopausal women. The quantitative analysis demonstrated that physical exercise led to an increase in HDL-C levels in perimenopausal women. In contrast, for obese postmenopausal women, 16 weeks of APT increased HDL-C levels and reduced total cholesterol and LDL-C levels, whereas 12 weeks of resistance exercise increased HDL-C levels and decreased triglycerides and LDL-C levels.

Only three of the included studies involved perimenopausal women (23,24,43). One of these studies evaluated the effects of aerobic exercise in dyslipidemic women and demonstrated positive changes in their lipid profiles (24), which has already been confirmed by other authors (11,53-57). Possible mechanisms that explain these results include increased basal metabolic rate, fatty acid absorption at the muscular level, activation of β-oxidation pathways, as well as glucose uptake and absorption. Additionally, this type of exercise has been shown to improve lipid metabolism by increasing the activity of antioxidant enzymes and reducing lipid peroxidation metabolites (58,59).

Studies involving healthy perimenopausal women did not report significant changes in lipid profiles after the intervention (23,43). One possible explanation for this is that the baseline parameters in the mentioned studies were within the normal range, which might have made any changes in lipid levels less significant (60). The meta-analysis conducted in this population showed an increase in HDL-C levels after physical exercise. However, these results should be interpreted with caution, considering the low number of included studies as well as their heterogeneity regarding the type of training and health conditions of the analyzed women (23,24). Further studies should be conducted on perimenopausal women with and without metabolic alterations to determine the effects of different exercise modalities on lipid profiles and long-term cardiovascular risk reduction.

Twenty-seven trials investigated the effects of exercise on the lipid profile of postmenopausal women (25-42,44-52). Three studies found an association between APT and an increase in HDL-C levels (27,35,41). This effect is well-established in the literature and can be explained by increased activity and lipoprotein lipase concentration in skeletal muscle (61-65). Lipoprotein lipase promotes the hydrolysis of triglycerides, releasing components such as cholesterol and phospholipids, which are essential for the maturation of HDL-C particles (66). Furthermore, recent evidence indicates that this type of training can enhance the antioxidant, anti-inflammatory, and antithrombotic effects of HDL-C by increasing nitric oxide availability and improving insulin resistance, factors that are relevant for reducing cardiovascular risk in this population (61, 66-68-).

The duration of APT programs varied widely, ranging from eight to twenty-four weeks. This aspect is important because more or less significant effects may be observed on the lipid profile depending on exercise duration and intensity (11). Our meta-analyses revealed that eight weeks of APT did not significantly alter the lipid profile, whereas 16 weeks of APT increased HDL-C levels and reduced total cholesterol and LDL-C levels in postmenopausal women. Possible mechanisms involved in the observed effects include: increased lipoprotein lipase activity, which not only regulates HDL-C levels but also enhances LDL-C hydrolysis (10,69), increased activity of reverse cholesterol transport, resulting in greater return of this lipid to the liver (10), improved insulin sensitivity, associated with greater lipid uptake at the muscular level and a decrease in hepatic lipogenesis, and finally increased mitochondrial density, which enhances the muscle capacity to oxidize fatty acids (70).

Concerning the studies investigating the effects of a 24-week walking program on the lipid profile of postmenopausal women, Ready et al. (31) observed a reduction in total cholesterol and triglyceride levels, while Dash et al. (35) reported an increase in HDL-C levels. Previous studies have also demonstrated the positive effects of a walking program on the lipid profile of this population (71-73). However, the meta-analysis conducted in this review did not show significant effects on serum lipid levels after 24 weeks of walking. This finding may be limited by the low number of studies included in the meta-analysis. Therefore, further studies should be conducted to clarify the effects of walking on the lipid profile of this population.

From another perspective, our meta-analysis showed that 12 weeks of resistance exercise training increased HDL-C levels and decreased triglyceride and LDL-C levels in obese postmenopausal women. These findings are consistent with previous studies conducted on women with similar profiles (12,74,75). The benefits are associated with reduced adipose tissue (33,76) and positive regulation of enzymes involved in lipolysis, such as adipocyte triacylglycerol lipase, hormone-sensitive lipase, and monoacylglycerol lipase (12,76). Resistance exercise also increases muscle strength and exercise capacity and improves body composition and quality of life (77). Therefore, postmenopausal women should be encouraged to engage in resistance exercise.

Finally, studies evaluating the effects of combined training (APT and resistance training) found that levels of HDL-C were higher after the intervention in postmenopausal women (29,30). In this regard, a network meta-analysis revealed that combined training had a greater effect on triglycerides and HDL-C levels in postmenopausal women (7). The possible mechanisms to increase serum HDL-C levels after aerobic and resistance exercises have been previously described. Therefore, combined training may result in a combination of effects. However, the mechanisms of these effects still need to be fully identified.

## Strengths and limitations

This review has strengths and limitations that should be considered when interpreting the results. As strengths, to the best of our knowledge, this is the first systematic review with meta-analysis to synthesize the effects of physical exercise on the lipid profile of climacteric women. Furthermore, our review included only randomized clinical trials with specific criteria to define the climacteric phase (perimenopause or postmenopause), which strengthens our results by ensuring that the groups of women analyzed are in comparable phases. Additionally, our meta-analyses involving postmenopausal women were highly specific, combining studies with similar populations and interventions. This is particularly important because postmenopausal women often present obesity and metabolic disorders that can interfere with the lipid profile and consequently affect the results.

As limitations, we must report the reduced number of participants in the analyzed clinical trials, the lack of control over confounding factors (such as dietary habits), and the moderate methodological quality determined by the nature of the studies themselves, which does not allow the use of placebos. Additionally, the selection criterion for analysis based on the “intervention protocol” rather than on the “intention-to-treat” may lead to bias in the results. Moreover, given the limited number of trials included in the meta-analysis, statistical heterogeneity should be interpreted with caution (78).

## Clinical practice and future research

Our results suggested that regular physical exercise is an important non-pharmacological tool for improving the lipid profile and consequently reducing the cardiovascular risk of perimenopausal and postmenopausal women. Specifically, 16 weeks of APT and 12 weeks of resistance training should be considered for regulating the lipid profile of obese postmenopausal women. However, further studies are needed to strengthen the level of evidence and determine the effects of different types and intensities of physical exercise on the lipid profile of this population, particularly perimenopausal women. Future clinical trials should include a larger number of participants, control for confounding factors, and adopt more rigorous and controlled designs to confirm and expand these findings.

## Conclusions

In summary, the studies suggested that physical exercise has a positive impact on the lipid profile of perimenopausal and postmenopausal women. Specifically, 12 weeks of resistance exercise and 16 weeks of APT have demonstrated beneficial effects in postmenopausal women, particularly obese women. However, there was limited evidence regarding the effects of different exercise modalities on the lipid profile of perimenopausal women, highlighting the need for further research involving this population.

## Supplementary Material

Click to view [pdf].
