# Supplementary material for: Effects of physical exercise on the lipid profile of perimenopausal and postmenopausal women: a systematic review and meta-analysis
Source: Braz J Med Biol Res. 2025 Mar 3;58:e14194. doi: 10.1590/1414-431X2025e14194 (PMC11884766; doi:10.1590/1414-431X2025e14194)
Supplement: Supplementary file 1 [file 1414-431X-bjmbr-58-e14194-suppl.pdf]

Table S1. PRISMA 2009 checklist (Moher et al. doi: 10.1371/journal.pmed.1000097).

Section/topic	#	Checklist item	Reported on page #
**Title**			
Title	1	Identify the report as a systematic review, meta-analysis, or both.	1
**Abstract**			
Structured summary	2	Provide a structured summary including, as applicable: background; objectives; data sources; study eligibility criteria, participants, and interventions; study appraisal and synthesis methods; results; limitations; conclusions and implications of key findings; systematic review registration number.	2
**Introduction**			
Rationale	3	Describe the rationale for the review in the context of what is already known.	3
Objectives	4	Provide an explicit statement of questions being addressed with reference to participants, interventions, comparisons, outcomes, and study design (PICOS).	3
**Methods**			
Protocol and registration	5	Indicate if a review protocol exists, if and where it can be accessed (e.g., Web address), and, if available, provide registration information including registration number.	4
Eligibility criteria	6	Specify study characteristics (e.g., PICOS, length of follow-up) and report characteristics (e.g., years considered, language, publication status) used as criteria for eligibility, giving rationale.	4
Information sources	7	Describe all information sources (e.g., databases with dates of coverage, contact with study authors to identify additional studies) in the search and date last searched.	4
Search	8	Present full electronic search strategy for at least one database, including any limits used, such that it could be repeated.	4
Study selection	9	State the process for selecting studies (i.e., screening, eligibility, included in systematic review, and, if applicable, included in the meta-analysis).	4,5
Data collection process	10	Describe method of data extraction from reports (e.g., piloted forms, independently, in duplicate) and any processes for obtaining and confirming data from investigators.	5
Data items	11	List and define all variables for which data were sought (e.g., PICOS, funding sources) and any assumptions and simplifications made.	5
Risk of bias in individual studies	12	Describe methods used for assessing risk of bias of individual studies (including specification of whether this was done at the study or outcome level), and how this information is to be used in any data synthesis.	5
Summary measures	13	State the principal summary measures (e.g., risk ratio, difference in means).	5
Synthesis of results	14	Describe the methods of handling data and combining results of studies, if done, including measures of consistency (e.g., I^2) for each meta-analysis.	5
**Section/topic**	**#**	**Checklist item**	**Reported on page #**
Risk of bias across studies	15	Specify any assessment of risk of bias that may affect the cumulative evidence (e.g., publication bias, selective reporting within studies).	5
Additional analyses	16	Describe methods of additional analyses (e.g., sensitivity or subgroup analyses, meta-regression), if done, indicating which were pre-specified.	n/a
**Results**			
Study selection	17	Give numbers of studies screened, assessed for eligibility, and included in the review, with reasons for exclusions at each stage, ideally with a flow diagram.	6 (see Figure 1)
Study characteristics	18	For each study, present characteristics for which data were extracted (e.g., study size, PICOS, follow-up period) and provide the citations.	6-13 (see Tables 3 and 4)
Risk of bias within studies	19	Present data on risk of bias of each study and, if available, any outcome level assessment (see item 12).	6 (see Tables 1 and 2)
Results of individual studies	20	For all outcomes considered (benefits or harms), present, for each study: (a) simple summary data for each intervention group (b) effect estimates and confidence intervals, ideally with a forest plot.	6-13 (see Tables 3 and 4)
Synthesis of results	21	Present results of each meta-analysis done, including confidence intervals and measures of consistency.	7–11 (see Figures 2,3,4,5, and 6)
Risk of bias across studies	22	Present results of any assessment of risk of bias across studies (see Item 15).	6
Additional analysis	23	Give results of additional analyses, if done (e.g., sensitivity or subgroup analyses, meta-regression [see Item 16]).	n/a
**Discussion**			
Summary of evidence	24	Summarize the main findings including the strength of evidence for each main outcome; consider their relevance to key groups (e.g., healthcare providers, users, and policy makers).	14–17
Limitations	25	Discuss limitations at study and outcome level (e.g., risk of bias), and at review-level (e.g., incomplete retrieval of identified research, reporting bias).	16,17
Conclusions	26	Provide a general interpretation of the results in the context of other evidence, and implications for future research.	17,18
**Funding**			
Funding	27	Describe sources of funding for the systematic review and other support (e.g., supply of data); role of funders for the systematic review.	18

Table S2. Search strategy.

Database	Search strategy
PubMed	("Climacteric"[Mesh] OR "Perimenopause"[Mesh] OR "Menopause"[Mesh] OR "Menopause, Premature"[Mesh] OR "Postmenopause"[Mesh]) AND ("Exercise"[Mesh] OR "Exercise Therapy"[Mesh] OR "exercise training" OR "physical exercise" OR "Resistance Training"[Mesh] OR "Endurance Training"[Mesh]) AND ("Lipoproteins, LDL"[Mesh] OR "Lipoproteins, VLDL"[Mesh] OR "Cholesterol"[Mesh] OR "Triglycerides"[Mesh] OR "Hyperlipidemias"[Mesh] OR "Hypercholesterolemia"[Mesh] OR "Dyslipidemias"[Mesh]) AND (“Clinical Trial”[PT] OR “Comparative Study”[PT] OR “Clinical Trials as Topic”[MeSH] OR random*[TIAB] OR controll*[TIAB] OR “intervention study”[TIAB] OR “experimental study”[TIAB] OR trial[TIAB] OR trials[TIAB] OR evaluat*[TIAB] OR repeat*[TIAB] OR compar*[TIAB]) NOT(“Animals”[MeSH] NOT (Animals[MeSH] AND “Humans”[MeSH]))
EMBASE	('climacterium'/exp OR 'climacteric' OR 'climacterium' OR 'menopausal transition' OR 'perimenopausal female' OR 'perimenopausal woman' OR 'perimenopause' OR 'menopausal age' OR 'menopause'/exp OR 'postmenopause'/exp OR 'post menopause' OR 'postmenopausal female' OR 'postmenopausal period' OR 'postmenopausal women' OR 'postmenopause') AND ('exercise'/exp OR 'biometric exercise' OR 'effort' OR 'exercise' OR 'exercise training' OR 'exertion' OR 'fitness training' OR 'fitness workout' OR 'physical effort' OR 'physical exercise' OR 'physical exertion' OR 'physical work-out' OR 'physical workout' OR 'exercise therapy' OR 'resistance training'/exp OR 'resistance exercise' OR 'resistance exercise training' OR 'resistance training' OR 'resistance-type exercise' OR 'resistance-type training' OR 'strength training' OR 'strength-type exercise' OR 'strength-type training' OR 'endurance training'/exp OR 'endurance exercise' OR 'endurance exercise training' OR 'endurance training' OR 'endurance workout' OR 'endurance-type exercise' OR 'endurance-type training') AND ('hyperlipidemia'/exp OR 'hyperlipaemia' OR 'hyperlipemia' OR 'hyperlipidaemia' OR 'hyperlipidaemia type ii' OR 'hyperlipidaemia type iii' OR 'hyperlipidaemia type v' OR 'hyperlipidaemias' OR 'hyperlipidemia' OR 'hyperlipidemias' OR 'hyperlipidemic' OR 'lipemia' OR 'lipidaemia' OR 'lipidemia' OR 'hypercholesterolemia'/exp OR 'cholesteremia' OR 'cholesterinemia' OR 'cholesterolemia' OR 'hypercholesteremia' OR 'hypercholesterinaemia' OR 'hypercholesterinemia' OR 'hypercholesterolaemia' OR 'hypercholesterolemia' OR 'dyslipidemia'/exp OR 'dyslipaemia' OR 'dyslipemia' OR 'dyslipidaemia' OR 'dyslipidaemias' OR 'dyslipidemia' OR 'dyslipidemias' OR 'lipidaemia, dys' OR 'lipidemia, dys' OR 'lipoprotein'/exp OR 'lipopolypeptide' OR 'lipoprotein' OR 'lipoprotein complex' OR 'lipoprotein polypeptide' OR 'lipoproteins' OR 'cholesterol'/exp OR 'triacylglycerol'/exp OR 'fatty acid triglyceride' OR 'triacylglycerol' OR 'triglyceride' OR 'triglycerides' OR 'tryglyceride' OR 'lipid'/exp OR 'lipids' OR 'low density lipoprotein cholesterol'/exp OR 'ldl cholesterol' OR 'cholesterol, ldl' OR 'lipoproteins, ldl cholesterol' OR 'low density lipoprotein cholesterol' OR 'high density lipoprotein cholesterol'/exp OR 'hdl cholesterol' OR 'cholesterol, hdl' OR 'high density lipoprotein cholesterol' OR 'lipoproteins, hdl cholesterol' OR 'very low density lipoprotein cholesterol'/exp OR 'cholesterol, vldl' OR 'lipoproteins, vldl cholesterol' OR 'very low density lipoprotein cholesterol' OR 'vldl cholesterol') AND ('clinical trial'/exp OR 'clinical trial' OR 'major clinical trial' OR 'trial, clinical' OR 'controlled clinical trial'/exp OR 'clinical trial, controlled' OR 'controlled clinical comparison' OR 'controlled clinical experiment' OR 'controlled clinical study' OR 'controlled clinical test' OR 'controlled clinical trial' OR 'randomized controlled trial'/exp OR 'controlled trial, randomized' OR 'randomised controlled study' OR 'randomised controlled trial' OR 'randomized controlled study' OR 'randomized controlled trial' OR 'trial, randomized controlled') AND ([randomized controlled trial]/lim OR 'controlled clinical trial'/de) AND ([article]/lim OR [article in press]/lim) AND ([english]/lim OR [portuguese]/lim OR [spanish]/lim) AND [female]/lim AND [humans]/lim
Web of Science	Search: #1 AND #2 AND #3 AND #4 AND #5
	#5: DT=(Article)
	#4: (((((((((((((ALL=(“Clinical Trial”)) OR ALL=(“Comparative Study”)) OR ALL=(“Clinical Trials as Topic”)) OR ALL=(random*)) OR ALL=(controll*)) OR ALL=(“intervention study”)) OR ALL=(“experimental study”)) OR ALL=(trial)) OR ALL=(trials)) OR ALL=(evaluat*)) OR ALL=(repeat*)) OR ALL=(compar*)) NOT ALL=(Animals)) NOT ALL=(Animals AND Humans)
	#3: ((((((ALL=("Lipoproteins, LDL")) OR ALL=("Lipoproteins, VLDL")) OR ALL=(Cholesterol)) OR ALL=(Triglycerides)) OR ALL=(Hyperlipidemias)) OR ALL=(Hypercholesterolemia)) OR ALL=(Dyslipidemias)
	#2: (((((ALL=(Exercise)) OR ALL=("Exercise Therapy")) OR ALL=("exercise training")) OR ALL=("physical exercise")) OR ALL=("Resistance Training")) OR ALL=("Endurance Training")
	#1: ((((ALL=(Climacteric)) OR ALL=(Perimenopause)) OR ALL=(Menopause)) OR ALL=("Menopause, Premature")) OR ALL=(Postmenopause)
Scopus	(TITLE-ABS-KEY ("Climacteric") OR TITLE-ABS-KEY ("Perimenopause") OR TITLE-ABS-KEY ("Menopause") OR TITLE-ABS-KEY ("Menopause, Premature") OR TITLE-ABS -KEY ("Postmenopause") AND TITLE-ABS-KEY ("Exercise") OR TITLE-ABS-KEY ("Exercise Therapy") OR TITLE-ABS-KEY ("exercise training") OR TITLE-ABS -KEY ("physical exercise") OR TITLE-ABS-KEY ("Resistance Training") OR TITLE-ABS-KEY ("Resistance Training") AND TITLE-ABS-KEY ("Lipoproteins, LDL") OR TITLE- ABS-KEY ("Lipoproteins, VLDL") OR TITLE-ABS-KEY ("Cholesterol") OR TITLE-ABS-KEY ("Triglycerides") OR TITLE-ABS-KEY ("Hyperlipidemias") OR TITLE-ABS-KEY ("Hypercholesterolemia") OR TITLE-ABS-KEY ("Dyslipidemias") AND TITLE-ABS-KEY ("Clinical Trial") OR TITLE-ABS-KEY ("Comparative study") OR TITLE-ABS-KEY ("Clinical trials as topic") OR TITLE-ABS-KEY ("random") OR TITLE-ABS-KEY ("control") OR TITLE-ABS-KEY ("intervention study") OR TITLE-ABS-KEY ("experimental study") OR TITLE-ABS-KEY ("trial") OR TITLE-ABS-KEY ("tests") OR TITLE-ABS-KEY ("evaluation") OR TITLE-ABS-KEY ("repeat") OR TITLE-ABS-KEY ("compare") AND NOT TITLE-ABS-KEY ("Animals") AND NOT TITLE-ABS-KEY ("Animals" AND "Humans")) AND (LIMIT-TO (DOCTYPE , "ar"))

Table S3. Original data from studies about lipid levels (mmol/L) before and after the intervention.

Trial	Intervention group			Control group		
	Baseline	After	Change	Baseline	After	Change
Elliott et al. (2002)[1,a]	6.94 ± 1.66	6.50 ± 1.22	Not reported	6.62 ± 1.21	6.77 ± 1.37	Not reported
Elliott et al. (2002)[2,a]	1.81 ± 1.54	1.68 ± 0.76	Not reported	1.53 ± 0.80	1.92 ± 1.14	Not reported
Elliott et al. (2002)[3,a]	1.99 ± 0.37	2.01 ± 0.32	Not reported	1.85 ± 0.38	1.96 ± 0.37	Not reported
Elliott et al. (2002)[4,a]	4.23 ± 1.69	4.12 ± 1.35	Not reported	4.45 ± 0.82	4.43 ± 1.52	Not reported
Miyaki et al. (2012)[1,a]	5.79 ± 0.83	5.97 ± 0.7	Not reported	5.72 ± 0.52	5.87 ± 0.62	Not reported
Miyaki et al. (2012)[2,a]	1.17 ± 0.45	1.06 ± 0.48	Not reported	1.24 ± 0.59	1.37 ± 0.58	Not reported
Miyaki et al. (2012)[3,a]	1.68 ± 0.28	1.86 ± 0.31	Not reported	1.68 ± 0.39	1.68 ± 0.34	Not reported
Miyaki et al. (2012)[4,a]	3.57 ± 0.72	3.67 ± 0.62	Not reported	3.47 ± 0.39	3.49 ± 0.44	Not reported
Ward et al. (2020)[1,c,#]	5.6 (4.8; 6.6)	5.3 (4.4; 6.5)	Not reported	6.2 (5.7; 6.8)	6.0 (5.5; 7.0)	Not reported
Ward et al. (2020)[2,c,#]	1.0 (0.7; 1.7)	1.2 (0.8; 1.4)	Not reported	0.9 (0.7; 1.4)	0.9 (0.8; 1.6)	Not reported
Ward et al. (2020)[3,c,#]	1.8 (1.5; 2.2)	1.8 (1.6; 2.4)	Not reported	1.8 (1.6; 2.5)	2.2 (1.5; 2.6)	Not reported
Ward et al. (2020)[4,c,#]	2.9 (2.6; 4.1)	2.9 (2.4; 4.1)	Not reported	3.6 (3.2; 4.2)	3.8 (3.0; 4.3)	Not reported
Wooten et al. (2011)[1,b,*]	5.31 ± 0.40	4.26 ± 0.41	Not reported	5.44 ± 0.32	5.89 ± 0.64	Not reported
Wooten et al. (2011)[2,b,*]	1.09 ± 0.05	1.04 ± 0.14	Not reported	1.14 ± 0.16	1.34 ± 0.16	Not reported
Wooten et al. (2011[3,b,*]	1.42 ± 0.14	1.34 ± 0.13	Not reported	1.46 ± 0.10	1.59 ± 0.12	Not reported
Wooten et al. (2011)[4,b,*]	3.39 ± 0.33	2.45 ± 0.30	Not reported	3.46 ± 0.29	3.69 ± 0.57	Not reported
Ready et al. (1995)[1,a]	6.64 ± 0.49	6.34 ± 0.56	Not reported	6.74 ± 0.59	6.75 ± 0.49	Not reported
Ready et al. (1995)[2,a]	1.80 ± 0.99	1.68 ± 0.93	Not reported	1.74 ± 0.56	1.91 ± 0.56	Not reported
Ready et al. (1995)[3,a]	1.35 ± 0.33	1.35 ± 0.37	Not reported	1.22 ± 0.16	1.15 ± 0.18	Not reported
Ready et al. (1995)[4,a]	4.53 ± 0.75	4.34 ± 0.69	Not reported	4.85 ± 0.64	4.93 ± 0.56	Not reported
Pereira et al. (2020)[1,a]	5.6 ± 0.8	5.5 ± 0.8	−0.2 ± 0.5	5.4 ± 1.0	5.2 ± 1.0	−0.2 ± 0.4
Pereira et al. (2020)[2,a]	1.2 ± 0.4	1.1 ± 0.4	−0.1 ± 0.4	1.1 ± 0.5	1.0 ± 0.5	−0.1 ± 0.4
Pereira et al. (2020)[3,a]	1.5 ± 0.4	1.5 ± 0.4	−0.0 ± 0.2	1.5 ± 0.4	1.5 ± 0.4	0.0 ± 0.2
Pereira et al. (2020)[4,a]	3.6 ± 0.9	3.4 ± 0.8	−0.1 ± 0.4	3.4 ± 0.9	3.2 ± 0.8	−0.2 ± 0.3

The data are presented in mmol/L. [1]Total cholesterol; [2]triglyceride; [3]high-density lipoprotein cholesterol; [4]low-density lipoprotein cholesterol; [a]data are reported as means±SD; [b]data are reported as means±SE; [c]data are reported as median and interquartile range; *data after 24 h; [#]data from the group with good compliance. References: Elliott KJ et al. doi: 10.1136/bjsm.36.5.340. Miyaki A et al. doi: 10.1139/h2012-069. Pereira R et al. doi: 10.1016/j.pcad.2020.10.005. Ready AE et al. PMID: 7489529. Ward LJ et al. doi: 10.1038/s41598-020-60759-z. Wooten JS et al. doi: 10.1055/s-0030-1268008.

Table S4. Standardized data of lipid profile concentrations (mg/dL) before and after the intervention.

Trial	Intervention group			Control group		
	Baseline	After	Change	Baseline	After	Change
Elliott et al. (2002)[1,a]	267.95 ± 64.09	250.96 ± 47.10	Not reported	255.60 ± 46.72	261.39 ± 52.89	Not reported
Elliott et al. (2002)[2,a]	160.18 ± 136.28	148.67 ± 62.26	Not reported	135.40 ± 70.80	169.91 ± 100.88	Not reported
Elliott et al. (2002)[3,a]	76.83 ± 14.28	77.61 ± 12.35	Not reported	71.43 ± 14.67	75.67 ± 14.28	Not reported
Elliott et al. (2002)[4,a]	163.32 ± 65.25	159.07 ± 52.12	Not reported	171.81 ± 61.66	171.04 ± 58.69	Not reported
Miyaki et al. (2012)[1,a]	223.55 ± 32.05	230.50 ± 27.02	Not reported	220.85 ± 20.07	226.64 ± 23.94	Not reported
Miyaki et al. (2012)[2,a]	103.54 ± 39.82	93.81 ± 42.48	Not reported	109.73 ± 52.21	121.24 ± 51.33	Not reported
Miyaki et al. (2012)[3,a]	64.86 ± 10.81	71.81 ± 11.97	Not reported	64.86 ± 15.05	64.86 ± 13.13	Not reported
Miyaki et al. (2012)[4,a]	137.84 ± 27.80	141.70 ± 23.94	Not reported	133.98 ± 15.06	134.75 ± 16.99	Not reported
Ward et al. (2020)[1,c,#]	216 (185; 255)	204 (170; 251)	Not reported	239 (220; 262)	231 (212; 270)	Not reported
Ward et al. (2020)[2,c,#]	88 (62; 150)	106 (71; 124)	Not reported	80 (62; 124)	79 (71; 141)	Not reported
Ward et al. (2020)[3,c,#]	69 (58; 85)	69 (62; 92)	Not reported	69 (62; 96)	85 (58; 100)	Not reported
Ward et al. (2020)[4,c,#]	112 (100; 158)	112 (93; 158)	Not reported	139 (123; 162)	147 (116; 166)	Not reported
Wooten et al. (2011)[1,b,*]	205.01 ± 15.44	164.48 ± 15.83	Not reported	210.04 ± 12.36	227.41 ± 24.71	Not reported
Wooten et al. (2011)[2,b,*]	96.46 ± 4.42	92.04 ± 12.39	Not reported	100.88 ± 14.16	118.58 ± 14.16	Not reported
Wooten et al. (2011)[3,b,*]	54.83 ± 5.40	51.74 ± 5.02	Not reported	56.37 ± 3.86	61.39 ± 4.63	Not reported
Wooten et al. (2011)[4,b,*]	130.89 ± 12.74	94.59 ± 11.58	Not reported	133.59 ± 11.20	142.47 ± 22.01	Not reported
Ready et al. (1995)[1,a]	256.37 ± 18.92	244.79 ± 21.62	Not reported	260.23 ± 22.78	260.61 ± 18.92	Not reported
Ready et al. (1995)[2,a]	159.29 ± 87.61	148.67 ± 82.30	Not reported	153.98 ± 49.56	169.03 ± 49.56	Not reported
Ready et al. (1995)[3,a]	52.12 ± 12.74	52.12 ± 14.28	Not reported	47.10 ± 6.18	44.40 ± 6.95	Not reported
Ready et al. (1995)[4,a]	174.90 ± 28.96	167.57 ± 26.64	Not reported	187.26 ± 24.71	190.35 ± 21.62	Not reported
Pereira et al. (2020)[1,a]	216.22 ± 30.89	212.35 ± 30.89	−7.72 ± 19.30	208.49 ± 38.61	200.77 ± 38.61	−7.72 ± 15.44
Pereira et al. (2020)[2,a]	106.19 ± 35.40	97.35 ± 35.40	−8.85 ± 35.40	97.35 ± 44.25	88.50 ± 44.25	−8.85 ± 35.40
Pereira et al. (2020)[3,a]	57.91 ± 15.44	57.91 ± 15.44	−0.00 ± 7.72	57.91 ± 15.44	57.91 ± 15.44	0.00 ± 7.72
Pereira et al. (2020)[4,a]	139.0 ± 34.75	131.27 ± 30.89	−3.86 ± 15.44	131.27 ± 34.75	123.55 ± 30.89	−7.72 ± 11.58

The data are presented in mg/dL. [1]Total cholesterol; [2]triglyceride; [3]high-density lipoprotein cholesterol; [4]low-density lipoprotein cholesterol; [a]data are reported as means±SD; [b]data are reported as means±SE; [c]data are reported as median and interquartile range; *data after 24 h; [#]data from the group with good compliance. References: Elliott KJ et al. doi: 10.1136/bjsm.36.5.340. Miyaki A et al. doi: 10.1139/h2012-069. Pereira R et al. doi: 10.1016/j.pcad.2020.10.005. Ready AE et al. PMID: 7489529. Ward LJ et al. doi: 10.1038/s41598-020-60759-z. Wooten JS et al. doi: 10.1055/s-0030-1268008.

Table S5. Methodological quality of the included trials (n=30).

	Methodological quality (PEDro Scale)												
First author	Eligibility criteria	Random assignment	Allocation concealment	Initial comparability	Participant blinding	Provider blinding	Blinding of outcome assessors	Retention	Analysis by intention to treat	Comparison between groups	Variability of key outcome provided	Total score	Quality of study
Blumenthal et al. (43)	Y	Y	Y	Y	N	N	N	Y	N	Y	Y	6	GOOD
Cardoso et al. (44)	Y	Y	Y	Y	N	N	N	N	N	Y	Y	6	GOOD
Cauley et al. (42)	Y	Y	Y	Y	N	N	N	Y	N	Y	Y	6	GOOD
Colado et al. (45)	Y	Y	Y	Y	N	N	N	N	N	Y	Y	6	GOOD
Conceição et al. (36)	Y	Y	Y	N	N	N	N	N	N	Y	Y	4	FAIR
Costa et al. (24)	Y	Y	Y	Y	N	N	Y	N	N	Y	Y	6	GOOD
Dash et al. (35)	Y	Y	Y	N	N	N	N	N	Y	Y	Y	5	FAIR
Diniz et al. (46)	Y	Y	Y	Y	N	N	N	N	N	Y	Y	6	GOOD
Elliott et al. (47)	N	Y	Y	N	N	N	N	N	N	Y	Y	4	FAIR
Gómez-Tomás et al. (28)	Y	Y	Y	Y	N	N	N	N	N	Y	Y	5	FAIR
Kazemi et al. (48)	Y	Y	Y	Y	N	N	N	N	N	Y	Y	7	GOOD
Krishnan et al. (23)	Y	Y	Y	Y	N	N	Y	N	N	Y	Y	5	FAIR
Kim et al. (49)	Y	Y	Y	Y	N	N	N	Y	Y	Y	Y	8	GOOD
Lee et al. (32)	Y	Y	Y	Y	Y	N	N	Y	N	Y	Y	7	GOOD
Lee et al. (38)	Y	Y	Y	Y	N	N	N	Y	N	Y	Y	6	GOOD
Libardi et al. (37)	Y	Y	Y	N	N	N	N	N	N	Y	Y	4	FAIR
Machado et al. (50)	Y	Y	Y	N	N	N	N	N	N	Y	Y	5	FAIR
Miyaki et al. (27)	Y	Y	Y	Y	N	N	N	N	N	Y	Y	5	FAIR
Neves et al. (26)	Y	Y	Y	Y	N	N	Y	N	N	Y	Y	6	GOOD
Pereira et al. (34)	Y	Y	Y	N	N	N	N	Y	N	Y	Y	5	FAIR
Ready et al. (31)	N	Y	Y	Y	N	N	N	N	N	Y	Y	5	FAIR
Reis et al. (51)	N	Y	Y	Y	N	N	N	N	N	Y	Y	5	FAIR
Rodrigo et al. (40)	Y	Y	Y	Y	N	N	N	Y	N	Y	Y	6	GOOD
Rossi et al. (25)	Y	Y	Y	Y	N	N	Y	N	N	Y	Y	6	GOOD
Rossi et al. (29)	Y	Y	Y	Y	N	N	N	N	N	Y	Y	5	FAIR
Rossi et al. (30)	Y	Y	Y	Y	N	N	N	N	N	Y	Y	5	FAIR
Son et al. (33)	N	Y	Y	Y	N	N	Y	N	N	Y	Y	6	GOOD
Wang et al. (41)	Y	Y	Y	Y	N	N	Y	Y	N	Y	Y	7	GOOD
Ward et al. (52)	Y	Y	Y	Y	N	N	N	N	N	Y	Y	6	GOOD
Wooten et al. (30)	Y	Y	Y	N	N	N	N	N	N	Y	Y	4	FAIR

N: no; Y: yes.

Table S6. Summary of findings and certainty assessment–intervention (exercise *vs* control).

Certainty Assessment							Number of Patients		Effect		Certainty	Importance
No. of studies	Study design	Risk of bias	Inconsistency	Indirectness	Imprecision	Other considerations	Exercise	Control	Relative (95%CI)	Absolute (95%CI)		
Effect of exercise on high-density lipoprotein-cholesterol (HDL-C) in perimenopausal women (follow-up: mean 24 weeks)												
2	randomized trials	not serious	serious[a]	not serious	serious[b]	none	38	30	–	4.89 (0.97 to 8.81)	⊕⊕◯◯ Low	IMPORTANT
Effect of aerobic exercise on triglycerides in obese postmenopausal women (follow-up: mean 16 weeks)												
2	randomized trials	not serious	serious[a]	not serious	serious[b]	none	30	33	–	-22.36 (–29.67 to –15.05)	⊕⊕◯◯ Low	IMPORTANT
Effect of aerobic exercise on low-density lipoprotein-cholesterol (LDL-C) in obese postmenopausal women (follow-up: mean 16 weeks)												
2	randomized trials	not serious	not serious	not serious	serious[b]	none	30	33	–	–17.86 (–25.97 to –9.75)	⊕⊕⊕◯ Moderate	IMPORTANT
Effect of aerobic exercise on HDL-C in obese postmenopausal women (follow-up: mean 16 weeks)												
2	randomized trials	not serious	not serious	not serious	serious[b]	none	30	33	–	3.88 (0.56 to 7.20)	⊕⊕⊕◯ Moderate	IMPORTANT
Effect of resistance exercise on triglycerides in obese postmenopausal women (follow-up: mean 12 weeks)												
3	randomized trials	not serious	not serious	not serious	serious[b]	none	38	41	–	-14.86 (–26.62 to –3.09)	⊕⊕⊕◯ Moderate	IMPORTANT
Effect of resistance exercise on LDL-C in obese postmenopausal women (follow-up: mean 12 weeks)												
2	randomized trials	not serious	serious[a]	not serious	serious[b]	none	27	29	–	–16.36 (28.05 to–4.67)	⊕⊕◯◯ Low	IMPORTANT
Effect of resistance exercise on High-Density Lipoprotein-Cholesterol (HDL-C) in obese postmenopausal women (follow-up: mean 12 weeks)												
3	randomized trials	not serious	serious[a]	not serious	serious[b]	none	38	41	–	4.20 (1.16 to 7.23)	⊕⊕◯◯ Low	IMPORTANT

CI: confidence interval. [a]Substantial heterogeneity (I^2: 50 to 90%). [b]Total population size or number of events is less than 400.

Table S7. Effects of physical training on the lipid profile of postmenopausal women.

Study (Country)	Participants' characteristics	Participants at baseline (n)		Age	Intervention (intensity)	Evaluated outcomes	Significant differences between groups
Rossi et al. 2017 (25) (Brazil)	Healthy	CON	n=20	62.8 ± 5.9	Did not participate in regular physical activity.	TG; Chol; LDL-C; HDL-C	After training, a difference was observed between the AT and CON groups regarding TG values. However, the difference ceased to exist after adjusting food intake. After 16 weeks, the analysis included Group CON (n=8), Group AT (n=8) and Group CT (n=15).
		AT	n=20	60.6 ± 7.9	Aerobic training (100% of the anaerobic threshold). D: 16; Ds: 50; F: 3.		
		CT	n=20	62.4 ± 5.1	Aerobic plus strength training [ST (established by the maximum repetition zone - 12 to 15); AT (100% of the anaerobic threshold)]. D: 16; Ds: 60; F: 3.		
Neves et al. 2017 (26) (Brazil)	Healthy	AT	n=32	57.7 ± 4.8	Did not participate in any supervised exercise.	Chol; LDL-C; HDL-C	After training, the EX group showed a significant reduction in HDL-C values. The analysis included Group CON (n=19) and Group EX (n=27).
		ST	n=32	58.6 ± 3.9	Functional training (established by the critical speed protocol). D: 16; F: 3		
Miyaki et al. 2012 (27) (Japan)	Healthy	CON	n=11	60 ± 7	Maintained normal habits.	TG; Chol; LDL-C; HDL-C	After training, the EX group showed a significant increase in HDL-C values. The analysis included Group CON (n=11) and Group EX (n=11).
		EX	n=11	60 ± 6	Aerobic training (60 to 75% of the maximum HR). D: 8; Ds: 30–45; F: 3–5.		
Blumenthal et al. 1991 (43) (USA)	Healthy	AT	n=12	47 ± 2	Aerobic training (70% of the HR reserve). D: 12; Ds: 50; F: 3.	TG; Chol; LDL-C; HDL-C; VLDL-C; APO	After training, the AT group showed a significant reduction in HDL-C values and an increase in APO A-I values. The analysis included Group AT (n=12) and Group ST (n=11).
		ST	n=11		Strength training (established by the maximum repetition zone – 12 to 15 repetitions). D: 12; Ds: 55; F: 2.		
Gómez-Tomás et al. 2018 (28) (Spain)	Healthy	CON	n=30	70.4 ± 5.4	Did not perform any training.	TG; Chol; LDL-C; HDL-C; VLDL-C	After training, the EX group significantly reduced Chol and LDL-C values. Furthermore, the comparison between groups shows significant differences after training regarding TG and VLDL-C values. In this case, the CON group presented higher TG and VLDL-C values than the EX group. The analysis included Group CON (n=20) and Group EX (n=18).
		EX	n=30	70.9 ± 4.4	Resistance training (subjective perception of effort established the progressive intensity of training). D: 48; Ds: 50; F: 3.		
Rossi et al. 2016 (29) (Brazil)	Overweight or obesity	CON	n=34	62.6 ± 5.9	Did not participate in regular physical activity.	Chol; LDL-C; HDL-C; Chol/HDL	After training, the CBT group significantly increased HDL-C values, and the AT group significantly reduced the Chol/HDL value. The analysis included Group CON (n=18), Group AT (n=15), and Group CBT (n=32).
		AT	n=35	60.5 ± 7.3	Aerobic training (100% of the critical speed protocol). D: 16; Ds: 52.		
		CBT	n=35	60.3 ± 6.1	Aerobic plus strength training (ST (established by the maximum repetition zone - 12 to 15); AT (100% of the critical speed protocol)). D: 16; Ds: 57.		
Rossi et al. 2018 (30) (Brazil)	One group (TG≥150) *	CON	n=35	63.4 ± 7.5	Did not participate in regular physical activity.	TG; Chol; LDL-C; HDL-C; Chol/HDL	After training, the CT and CTH groups significantly increased HDL-C values and reduced Chol/HDL values. The analysis included Group CON (n=10), Group AT (n=13), Group CT (n=16) and Group CTH (n=14).
		AT	n=35	61.8 ± 7.9	Aerobic training (100% of the critical speed protocol). D: 16; Ds: 50.		
		CT	n=35	61.4 ± 5.0	Aerobic plus strength training [ST (established by the maximum repetition zone - 12 to 15); AT (100% of the critical speed protocol)]. D: 16; Ds: 60.		
		CT*	n=35	ns	Aerobic plus strength training [ST (established by the maximum repetition zone - 12 to 15); AT (100% of the critical speed protocol)]. D: 16; Ds: 60.		

Ready et al. 1995 (31) (Canada)	Hypercholesterolemia	CON	n=16	62.0 ± 5.7	Maintained their sedentary lifestyle.	TG; Chol; LDL-C; HDL-C; VLDL-C; Chol/HDL; HDL/LDL	After the intervention, the EX group showed a reduction in Chol, Chol/HDL, and TG values, which were statistically different from the CON group. The analysis included Group CON (n=10) and Group EX (n=15).
Lee et al. 2021 (32) (South Korea)	Obesity	CON	n=12	57.5 ± 2.9	Maintained normal habits.	TG; Chol; LDL-C; HDL-C	After the intervention, the groups showed significant differences. In this context, the EX group showed a reduction in Chol and LDL-C values, while the CON group had no significant change. The analysis included Group CON (n=12) and Group EX (n=12).
		EX	n=12	56.0 ± 2.9	Taekwondo training - Kicking and stepping exercises, and Taekwondo aerobics (50 to 80% of maximum HR). D:16; Ds: 60; F: 5.		
Son et al. 2021 (33) (South Korea)	Obesity and metabolic syndrome	CON	n=21	68.2 ± 1.4	Did not participate in any supervised exercise.	TG; LDL-C; HDL-C	After training, the EX group significantly increased HDL-C values and reduced TG and LDL values. The analysis included Group CON (n=17) and Group EX (n=18).
		EX	n=21	68.2 ± 1.6	Resistance training (40–70% of 1 maximum repetition). D: 12; Ds: 60; F: 3.		
Pereira et al. 2020 (34) (Portugal)	Healthy	CON	n=29	68.3 ± 6.2	Maintained their usual level of physical activity.	TG; Chol; LDL-C; HDL-C; Chol/HDL; Chol/LDL; LDL/HDL	After the intervention, the EX group significantly reduced Chol and LDL-C values. The analysis included Group CON (n=26) and Group EX (n=41).
		EX	n=42		Handball-based exercises (76 ± 6% of the maximum HR). D: 16; Ds: 60; F: 2–3.		
Dash et al. 2018 (35) (USA)	Obesity and metabolic syndrome	CON	n=71	58.4 ± 5.3	Maintained their daily activities.	TG; HDL-C	The participants were divided into two large groups: A group with a family history of breast cancer and a group without a family history of breast cancer. Each large group had a CON group, an AT group, and an HBE group. Differences were only observed in women with a family history of breast cancer. In this population, after the intervention, the HBE group significantly reduced TG values compared to the CON group. Furthermore, after the intervention, the AT and HBE groups significantly increased HDL-C values compared to the CON group. The analysis included Group CON (n=55), Group AT (n=49), and Group HBE (n=52).
		AT	n=73	58.1 ± 5.1	Aerobic training (45 to 65% of VO_2 maximum). D: 24; Ds: 50; F: 3.		
		HBE	n=69	58.3 ± 4.7	Walking program (The intensity was 11 to 14 (moderate) on a 20-point perceived exertion scale). D: 24.		
Conceição et al. 2013 (36) (Brazil)	Healthy	CON	n=10	53.0 ± 5.7	Did not participate in any supervised exercise.	TG; HDL-C	No significant differences were observed. The analysis included Group CON (n=10) and Group EX (n=10).
		EX	n=10	53.4 ± 3.9	Resistance training (established by the maximum repetition zone - Training volume decreased as the intensity increased). D: 16; F: 3.		
Libardi et al. 2012 (37) (Brazil)	Healthy	CON	n=12	51.2 ± 6.4	Did not participate in any supervised exercise.	TG; Chol; LDL-C; HDL-C	After training, the EX group significantly reduced cholesterol and LDL-C values. After training, differences were observed between the groups regarding cholesterol and LDL-C values. The analysis included Group CON (n=12) and Group EX (n=12).
		EX	n=12	53.7 ± 3.6	Resistance training (established by the maximum repetition zone - Training volume decreased as the intensity increased). D: 16; F:3.		

Lee et al. 2012 (38) (South Korea)	Obesity	CON<br>EX	n=8<br>n=8	54.2 ± 2.9<br>54.7 ± 2.8	Did not participate in any supervised exercise.<br>Yoga - coordination exercises and integration of breathing with movement. D: 16; Ds: 60; F: 3.	TG; Chol; LDL-C; HDL-C	After the intervention, the EX group showed lower Chol values than the CON group. The analysis included Group CON (n=8) and Group EX (n=8).
Wooten et al. 2011 (39) (USA)	Obesity	CON<br>EX	n=12<br>n=12	67.0 ± 0.6<br>64.4 ± 0.7	Did not participate in any supervised exercise.<br>Resistance training (2 sets of 8 maximum repetitions and a 3rd until failure - If the participant performed more than 12 repetitions in the third series, the weight was increased in the following training). D: 12; F: 3.	TG; Chol; LDL-C; HDL-C; HDL3-C; non-HDL-C; Chol/HDL; LDL/HDL	After training, the EX group significantly reduced Chol, LDL-C, HDL3-C, and non-HDL-C values. Furthermore, after training, the CON and EX groups showed significant differences in Chol, LDL-C, HDL3-C, and non-HDL-C values; the EX group had lower values in both variables. The analysis included Group CON (n=12) and Group EX (n=9).
Rodrigo et al. 2008 (40) (Spain)	Healthy	CON<br>EX	n=23<br>n=40	53.1 ± 1.9<br>54.3 ± 2.5	General recommendations about exercise and nutrition.<br>Resistance training (In the first eight weeks: performed 8 to 10 exercises - 2 sets of 20 repetitions per minute - the number of sets and reps has increased linearly to increase training intensity). D:24; F: 2-3.	TG; Chol; LDL-C; HDL-C; APO	After the intervention, both the CON and EX groups significantly increased HDL-C values. The analysis included Group CON (n=22) and Group EX (n=37).
Wang et al. 2014 (41) (China)	Dyslipidemia and obesity	CON<br>EX	n=23<br>n=23	55.1 ± 7.8<br>56.9 ± 6.2	Maintained their customary lifestyle.<br>Aerobic training (60–80% of the HR reserve). D: 12; Ds: 50; F: 3	TG; HDL-C	After training, group EX significantly improved the HDL-C values. The analysis included Group CON (n=23) and Group EX (n=23).
Cauley et al. 1987 (42) (USA)	Healthy	CON<br>EX	n=115<br>n=114	57.4 ± 4.2<br>57.9 ± 4.0	Maintained normal habits.<br>Walking program (goal: for each individual to walk a minimum of 7 miles per week - group walking two sessions per week, and participants were encouraged to walk alone at least once weekly. D: 96.	TG; Chol; HDL-C	No significant differences were observed. The analysis included Group CON (n=104) and Group EX (n=100).
Cardoso et al. 2016 (44) (Brazil)	Healthy	CON<br>EX	n=14<br>n=14	54.9 ± 6<br>54.7 ± 8	Maintained their customary lifestyle.<br>Resistance training (In the first two weeks: adaptation - 1 series of 15 repetitions using a charge considered easy and a frequency of 3 times a week; Until the 12th week, the frequency increased to 5/week and the sets and charge also increased progressively). D: 12; F: 3–5	TG; HDL-C;	No significant differences were observed. The analysis included Group CON (n=12) and Group EX (n=11).
Colado et al. 2009 (45) (Spain)	Healthy	CON<br>AE<br>EB	n=11<br>n=22<br>n=22	52.9 ± 1.9<br>54.7 ± 2.0<br>54.0 ± 2.8	Maintained their customary lifestyle.<br>Resistance training was performed in water (controlled by the number of repetitions and perceived effort). The AE group used different types of aquatic resistance. D: 24; F: 2–5.<br>Resistance training was performed with elastic bands (controlled by the number of repetitions and perceived effort). The EB group adjusted their grip on the elastic bands to control the resistance. D: 24; F: 2–5.	TG; Chol; LDL-C; HDL-C; Chol/HDL	After training, the Group EB significantly improved the HDL-C values and the Chol/HDL. The analysis included Group CON (n=10), Group AE (n=15), and Group EB (n=21).
Diniz et al. 2016 (46) (Brazil)	Healthy	CON<br>AT	n=12<br>n=12	62.0 ± 7.8<br>60.2 ± 8.5	Maintained a sedentary lifestyle.<br>Aerobic training is performed on a running track on separate, nonconsecutive days (established by the critical speed protocol). D: 8; Ds: 50.	TG; Chol; LDL-C; HDL-C; LDL/HDL	After the intervention, the Group AT showed a significant decrease in LDL-C when compared with the Group CON. The analysis included Group CON (n=9) and Group AT (n=10).

Elliott et al. 2002 (47) (England)	Healthy	CON<br>EX	n=7<br>n=8	53 ± 3<br>58 ± 4	Maintained a sedentary lifestyle.<br>Resistance training (Three sets of 8 repetitions at 80% of 10RM for eight weeks and then eight weeks of detraining). D: 8: F: 3.	TG; Chol; LDL-C; HDL-C; Chol/HDL; Chol/LDL	No significant main effects for these values. The analysis included Group CON (n=7) and Group EX (n=8).
Kazemi et al. 2023 (48) (Iran)	Metabolic Syndrome	CON<br>ST	n=16<br>n=16	ns<br>ns	Maintained usual routine.<br>Resistance training (In the first four weeks: three sets with 8-10 repetitions of each exercise with an intensity of 75% of 1RM. After four weeks: 8 repetitions of each exercise with an intensity of 80% 1RM). D: 8: F: 3	TG; Chol; LDL-C; HDL-C	In both training groups, HDL-C levels significantly increased, while LDL-C, TG, and Chol levels significantly decreased after training. The analysis included Group CON (n=15), Group ST (n=15), and Group HIIT (n=15).
		AT	n=16	ns	High-intensity interval training (3 minutes of high-intensity training at 80–90% of their maximum heart rate, followed by moderate walking for 3 min at 55–65% of HRmax) D: 8; F: 3		
Kim et al. 2012 (49) (South Korea)	Obesity	CON<br>EX	n=15<br>n=15	54.5 ± 2.8<br>54.5 ± 2.8	Maintained their customary lifestyle.<br>Aerobic exercise (The intensity was initially set at 55–65% of maximum heart rate and was gradually increased by 5% every 4 weeks until 70–80%). D: 16; Ds: 60; F: 3	TG; Chol; LDL-C; HDL-C	After training, the EX group showed lower Chol values compared to the CON group. The analysis included Group CON (n=15) and Group EX (n=15).
Machado et al. 2021 (50) (Brazil)	Type 2 diabetes	HI	n=10	ns	High-intensity functional training (heart rate between 50-70% of maximum heart rate). D:8; Ds:60; F: 3	TG; Chol; LDL-C; HDL-C; Chol/HDL	After training, HDL-C and TG levels improved in Group HI, although a reduction in LDL-C was observed only in Group MI. The analysis included Group HI (n=6) and Group MI (n=10).
		MI	n=14	ns	Moderate-intensity functional training (heart rate above 70% of maximum heart rate). D:8; Ds:60; F: 3		
Reis et al. 2014 (51) (Brazil)	Healthy	CON<br>EX	n=11<br>n=22	62.2 ± 3.0<br>56.2 ± 7.1	Did not perform any systematic training.<br>Aquatic aerobic training (low to high intensity, according to the subjective perception of effort) D: 16; Ds: 50; F: 3	TG; Chol; LDL-C; HDL-C	No significant differences were observed. The analysis included Group CON (n=11) and Group EX (n=22).
Ward et al. 2020 (52) (Sweden)	Healthy	CON<br>EX	n=32<br>n=33	55.4 ± 5<br>55.7 ± 5.1	Maintained their customary lifestyle.<br>Resistance training was performed using resistance machines and body weight (six exercises on seated resistance machines and two body-weight exercises). D: 15; F: 3	TG; Chol; LDL-C; HDL-C; APO	After training, the Group EX with good compliance showed a reduction in Chol, LDL-C, and non-HDL-C values. The analysis included Group CON (n=21) and Group EX (n=21).

n: sample size at baseline; CON: control; EX: exercise; AT: aerobic training; ST: strength training; CT: concurrent training; CBT: combined training; HBE: home based exercise; AE, aquatic exercise; EB: elastic bands; HI: high-intensity combined training; MI: moderate-intensity combined training; *group with TG≥150; LV: low volume; HV: high volume; ns: not specified; HR: heart rate; D: duration of the intervention (weeks); Ds: duration of the exercise session (minutes); F: exercise frequency (times/week); TG: triglycerides; Chol: total cholesterol; LDL-C: low-density lipoprotein cholesterol; HDL-C: high-density lipoprotein cholesterol; HDL3-C: high-density lipoprotein cholesterol fraction 3; non-HDL-C: non-HDL-C cholesterol; Chol/HDL ratio between Chol/HDL; Chol/LDL ratio between Chol/LDL; HDL/LDL: ratio between HDL/LDL; LDL/HDL: ratio between LDL/HDL; VLDL-C: very-low-density lipoprotein; APO: Apolipoprotein.
